# Mycotic Aneurysm with Iliac Artery-Colonic Fistula

**DOI:** 10.1155/2022/3250749

**Published:** 2022-03-04

**Authors:** Le Viet Dung, Ma Mai Hien, Dang-Thi Bich Nguyet, Thieu-Thi Tra My, Nguyen Minh Duc

**Affiliations:** ^1^Radiology Center, Hanoi Medical University Hospital, Hanoi, Vietnam; ^2^Department of Radiology, Hanoi Medical University, Hanoi, Vietnam; ^3^Department of Radiology, Pham Ngoc Thach University of Medicine, Ho Chi Minh City, Vietnam

## Abstract

Although mycotic (infected) aneurysms are uncommon, they can affect any artery. The most frequently involved vessel is the aorta as well as femoral and cerebral arteries. A vascular-colonic fistula from infected aneurysms is even rarer, which remains a challenge for diagnosis and treatment. In this case report, we aimed to illustrate an 89-year-old man presenting initially with an aneurysm of the right common iliac artery. Forty days later, this lesion was infected and produced fresh blood in the rectum and sigmoid colon observed by colonoscopy. The final diagnosis of this case was a right common iliac artery aneurysm-colonic fistula due to infection. The patient was successfully diagnosed and treated with surgery at our hospital.

## 1. Introduction

A mycotic aneurysm is an uncommon disease caused by infection of the artery wall [1]. It is difficult to diagnose early due to its silent manifestation; therefore, an infected aneurysm is often treated when complications arise, such as rupture or fistula. For unruptured mycotic aneurysms, the mortality rate is 15%–50%, whereas the mortality rate for ruptured aneurysms is as high as 90% [1]. Concerning a vascular-colonic fistula secondary to an infected aneurysm, its unusual complication with gastrointestinal bleeding is a decisive sign of this condition. If mycotic aneurysms rupture into the bowel, patients are likely to suffer a massive hemorrhage, fulminant sepsis, or even a fatality [2]. With a leaking aneurysm, the clinical onset may be gastrointestinal hemorrhage lasting from several days to months [2].

Imaging features play an important role in the detection of infected aneurysms in suspected cases and, in confirmed cases, imaging modalities also help clinicians assess complications and vascular mapping before treatment [3]. Several imaging modalities are used in the diagnosis of mycotic aneurysms, such as Doppler ultrasonography, multidetector computed tomography, magnetic resonance imaging (MRI), and conventional angiography; however, CT angiography remains the first choice. Furthermore, blood culture and biomarkers for fungal antigens such as cryptococcus antigen, galactomannan, or 1,3-beta-D-glucan are quite crucial for the determination of etiology. In terms of management, antibiotic therapy, intervention, and surgery are most often used [4, 5]. In this article, we intended to depict a rare case of a mycotic aneurysm with a right common iliac artery-colonic fistula.

## 2. Case Report

**Figure 1:**
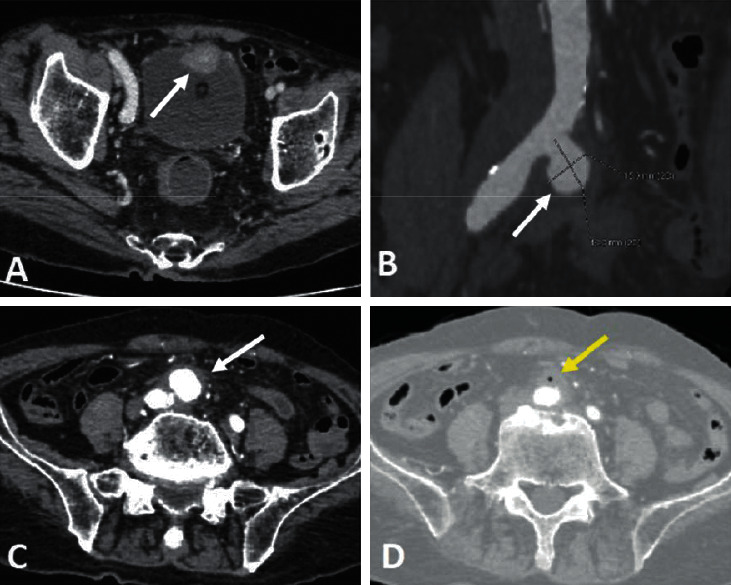
CT scanner forty days prior to admission. (a) Enhanced axial CT shows a bladder tumor (arrow). (b, c) Enhanced coronal and axial CT shows a 17 × 18 mm saccular aneurysm arising from the right common iliac artery (arrows) surrounded by fat stranding. (d) Enhanced axial CT shows small foci of gas (arrow).

An 89-year-old man presented to the emergency department with hematochezia for 7 days. His medical history was unremarkable. Forty days before admission, he had pain in his lower abdomen and hematuria. An abdominal CT scan showed a saccular aneurysm of the right common iliac artery (17 × 18 mm in size) with fat stranding, edema, gas, and prominent inflammatory soft tissue around the aneurysm (Figure 1) and a bladder tumor. He was treated with a complete resectioning of the bladder tumor, and the histopathology result demonstrated transitional cell carcinoma. However, the aneurysm was treated with conservative management.

A physical examination showed the patient was conscious with a temperature of 37.2°C, he had tachycardia with a pulse of 107 beats per minute, his blood pressure was 98/60 mmHg, his respiration was 16 beats per minute, and he had pale skin. An abdominal examination indicated his abdomen was soft without a palpable mass, hepatomegaly, or splenomegaly. A rectal examination showed hematochezia, but no other anomalies were detected. The laboratory test results were the following: hemoglobin 75 g/L, hematocrit 0.24 L/L, white blood cell 5.91 g/L, platelet 189 g/L, and C-reactive protein (CRP) 2.29 mg/dL.

**Figure 2:**
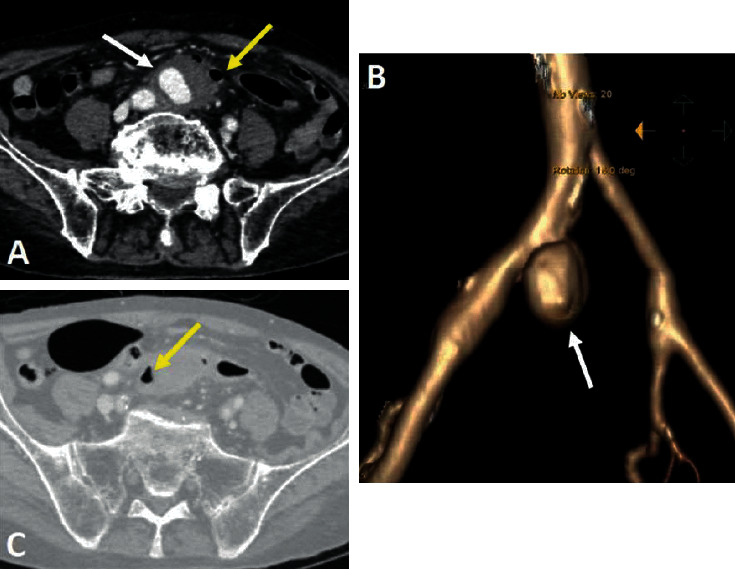
CT scanner at the time of admission. (a, b) Enhanced axial and reconstruction coronal CT shows a 31 × 36 × 40 mm saccular aneurysm (arrows) arising from the right common iliac artery and prominent inflammatory soft tissue around the aneurysm. The anterior wall of the aneurysm has adhered to the posterior wall of the sigmoid colon ((a), yellow arrow). Gas foci ((c), yellow arrow) were noted around the aneurysm.

**Figure 3:**
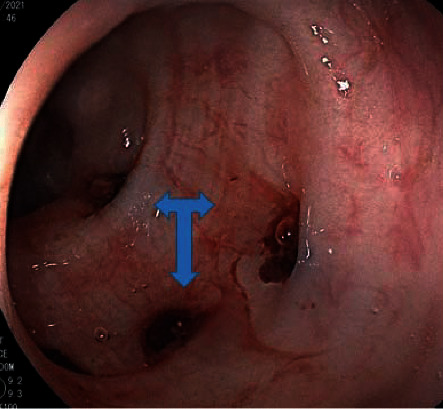
Colonoscopy revealed fresh blood (three-head arrow) in the rectum and sigmoid colon.

An abdominal CT scan at the time of admission showed a significant increase in the size of the aneurysm (31 × 36 × 40 mm), with a neck of 7 mm and irregular wall thickening with 15 mm of mural thrombus (Figure 2). The fat stranding, edema, gas, and inflammatory soft tissue surrounding the aneurysm were also increased compared to 40 days before, indicating that infectious process of aneurysm was increased. The anterior wall of the aneurysm had adhered to the posterior wall of the sigmoid colon, and a colonoscopy revealed fresh blood in the rectum and sigmoid colon and hematic infiltration of the sigmoid wall (Figure 3). The patient was diagnosed with a fistula between the mycotic right common iliac artery aneurysm and the sigmoid colon and underwent an emergency laparotomy.

**Figure 4:**
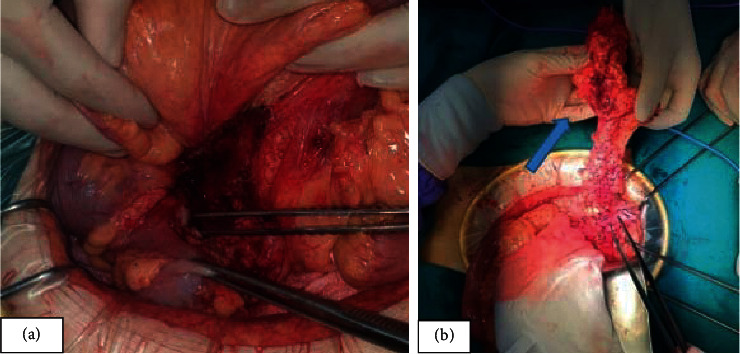
Intraoperative results (a, b); arrow in (b): injured sigmoid colon.

**Figure 5:**
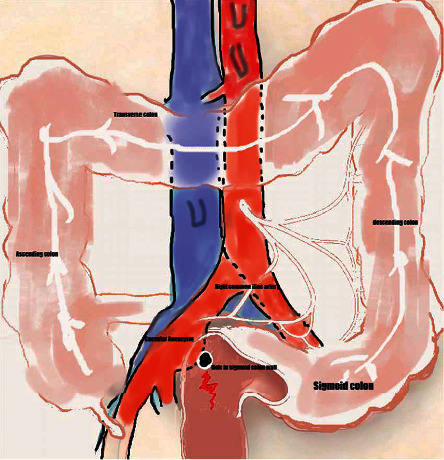
Illustration of the anatomic relations found at operation showing the right common iliac artery aneurysm, sigmoid colon, and the fistula between them.

Intraoperative results revealed a 40 mm saccular aneurysm of the right common iliac artery, of which the anterior wall had tightly adhered to the mesenteric edge of the sigmoid colon. The aneurysm had signs of inflammation: edema and adhesion on the surrounding tissue. A fistula between the sigmoid colon and aneurysm was determined on the anterior wall of the aneurysm with a diameter of about 30 mm (Figures 4 and 5). To diminish the risk of graft infection, the vascular surgeon decided to create a femoral-femoral bypass graft and then remove the aneurysm. A resectioning of the affected colon segment and end-colostomy (Hartmann's procedure) was performed. After surgery, the patient was given sulperazone and metronidazole. Two days later, he had a temperature of 39 °C. Subsequently, blood cultures were drawn, which grew *Escherichia coli* and *Enterococcus faecalis*. Imipenem, amikacin, and tigecycline were added to the antibiotic therapy. Fourteen days later, the patient's fever was resolved and his leukocyte count and CRP concentration were within normal ranges. He was discharged five days later.

## 3. Discussion

Mycotic aneurysms are saccular vascular dilatations caused by the destruction of the vessel wall by invasive organisms. It may result from hematogenous spread, lymphatic spread, or direct extension from an adjacent infected organ [6]. Mycotic aneurysms can involve any artery, but the aorta is the most frequently affected section [7]. The femoral artery is the most frequently involved peripheral artery due to intravenous drug abusers or interventional procedures, with mycotic iliac aneurysms presenting in about 2% of these cases [7].

A vascular-enteric fistula is a form of pathological communication between the vascular system and the gastrointestinal tract and may be the result of a myotic aneurysm. Aortoenteric fistulas can be classified as primary or secondary and most often involve the duodenum [8]. Fistulization of an iliac aneurysm into the sigmoid colon is rare and results in lower gastrointestinal hemorrhage. The clinical presentations are variable with acute or chronic, massive or slight gastrointestinal hemorrhage, such as hematochezia, hemorrhagic shock, or syncope [2]. These patients also suffer from pain related to the location of the lesion, such as abdominal, back, or suprapubic, and prolonged fever and sepsis may present related to infection. Some common pathogens of myotic aneurysms are *Streptococcus*, *Staphylococcus* and *Salmonella* [9]. Due to the high frequency of *Staphylococcus aureus*, it is essential to administer anti-methicillin-resistant *Staphylococcus aureus* drugs such as vancomycin, daptomycin, linezolid, sulfamethoxazole and trimethoprim (TMP-SMZ), quinupristin-dalfopristin, clindamycin, and tigecycline as empiric therapy [9, 10]. Gram-negative bacilli, including enterobacteria, are increasing and now comprise a major proportion of intra-abdominal infections [10].

Imaging modalities play a pivotal role in the early diagnosis, assessment, and treatment of mycotic aneurysms and their complications. A CT scan is the best diagnostic tool for the diagnosis, as the symptoms of a vascular-enteric fistula include effacement or blurred borders of fatty planes around arteries, soft tissue collection around the artery >5 mm, ectopic gas within or adjacent to the artery, intravenous contrast within the gastrointestinal lumen or around the artery, and intestinal wall thickening [2]. The presence of ectopic bowel gas or extravasation of contrast into the bowel lumen is 100% specific [11]. In this case, apart from the imaging features of an infected aneurysm, the CT scan also revealed that the anterior wall of the aneurysm had adhered to the posterior wall of the sigmoid colon. Associated with the symptoms of lower gastrointestinal bleeding, it strongly suggested a vascular-colonic fistula. Although some findings of a mycotic aneurysm may be noted on an MRI, such as mural edema and surrounding fat stranding, it is inferior to a CT scan because of the risk of artifact, small volume coverage, and unreliable detection of the presence of aortic extraluminal gas [3, 10]. Doppler ultrasonography in the diagnosis of an infected aneurysm is not as reliable as a CT scan or MRI and is limited to assessing deep structures. Angiography is rarely used as the first-line imaging modality; however, it may be helpful for surgical planning in certain cases or used to treat massive gastrointestinal bleeding secondary to vascular-enteric fistula by embolization [12]. Colonoscopy is also a useful modality for the diagnosis, as it may be useful in detecting hemorrhage and erosion of the intestinal wall [10].

Conservative management is associated with poor outcomes, including a mortality rate of 50% and a 1-year survival rate of only 32% [9]. Prompt diagnosis and timely treatment are necessary for patient recovery. Treatment management may include antibiotic therapy for controlling sepsis, surgery, and endovascular therapy [1, 5]. The operative technique or endovascular treatment depends on several factors, such as aneurysm location, the extent of the infection, the fitness of the patient, complications, and the surgeon's preference [5]. In this case, the operation was divided into two steps: vascular reconstruction and gastrointestinal repair. In the first step, to diminish the risk of graft infection, the vascular surgeon decided to create a bypass graft and remove the aneurysm. In the second step, the GI surgeon resected the sigmoid colon and formed a stoma in the lower-left quadrant.

## 4. Conclusion

A mycotic aneurysm is unusual, and its complication with iliac artery aneurysmo-colonic fistulas is even rarer. The clinical onset of this disease is unpredictable and can present with mild symptoms to life-threatening hemorrhage due to ruptured aneurysms. Imaging modalities are essential for the early diagnosis and assessment of mycotic aneurysms and their complications and, therefore, the treatment orientation of clinicians and surgeons.

## Data Availability

Data sharing is not applicable to this article as no datasets were generated or analysed during the current study.
